# Generic Dynamic Environment Perception Using Smart Mobile Devices

**DOI:** 10.3390/s16101721

**Published:** 2016-10-17

**Authors:** Radu Danescu, Razvan Itu, Andra Petrovai

**Affiliations:** Computer Science Department, Technical University of Cluj Napoca, 28 Memorandumului Street, Cluj Napoca 400114, Romania; razvan.itu@cs.utcluj.ro (R.I.); andra.petrovai@cs.utcluj.ro (A.P.)

**Keywords:** obstacle detection, occupancy grid, monocular vision, advanced driving assistance system, mobile device

## Abstract

The driving environment is complex and dynamic, and the attention of the driver is continuously challenged, therefore computer based assistance achieved by processing image and sensor data may increase traffic safety. While active sensors and stereovision have the advantage of obtaining 3D data directly, monocular vision is easy to set up, and can benefit from the increasing computational power of smart mobile devices, and from the fact that almost all of them come with an embedded camera. Several driving assistance application are available for mobile devices, but they are mostly targeted for simple scenarios and a limited range of obstacle shapes and poses. This paper presents a technique for generic, shape independent real-time obstacle detection for mobile devices, based on a dynamic, free form 3D representation of the environment: the particle based occupancy grid. Images acquired in real time from the smart mobile device’s camera are processed by removing the perspective effect and segmenting the resulted bird-eye view image to identify candidate obstacle areas, which are then used to update the occupancy grid. The occupancy grid tracked cells are grouped into obstacles depicted as cuboids having position, size, orientation and speed. The easy to set up system is able to reliably detect most obstacles in urban traffic, and its measurement accuracy is comparable to a stereovision system.

## 1. Introduction

Driving is a process of continuous sensing, processing the sensory information, making decisions and putting these decisions into action. Some parts of this loop can be done by humans, and some by machines. As all decisions related to driving are based on sensory information, sensing is maybe the most important part of the driving process. Humans are well equipped for sensing, but they are not perfect. This is why, for decades, researchers have tried to devise better sensors, and better and faster algorithms, to help perceive the environment, help the driver and increase traffic safety.

While most of the research and development effort has been dedicated to adding intelligent sensing capabilities to the vehicles themselves, especially to the higher end ones, driving assistance applications on mobile devices are a less costly alternative. Facilitated by the rapid evolution of the processing power of the smart mobile devices, and by the increasingly complex and accurate sensors that come embedded into these devices, driving assistance based on image and signal processing on mobile devices may quickly become a reliable tool for traffic safety.

This paper presents a method for perceiving the obstacles from the dynamic driving environment based on on-board, real time processing of the images from the camera of a smart mobile device (smartphone, and tablet) that can be fitted behind the windshield of a car. Besides the image sequence stream, the algorithms will use other sensors, such as the GPS receiver, the gyroscope, and the accelerometer to know the motion of the user’s car, and delimit this motion from the proper motion of the obstacles.

The method proposed in this paper relies on segmenting obstacle areas from an image, after the perspective effect is removed, followed by using these areas to update a probabilistic occupancy grid, which is able to filter out some detection errors, and extract dynamic properties for each grid cell. The estimated occupied grid cells, having also speed and orientation, are grouped into obstacles. In this way, no assumption about the nature or shape of the obstacle is made, and detected obstacles that are the system’s result will have position, size, speed and orientation assigned to them.

## 2. Related Work

The set of driving assistance applications available includes iOnRoad, Drivea, and Movon FCW. iOnRoad [1] is one of the first augmented driving solutions available for iOS and Android, using the smartphone’s camera and GPS receiver to provide time to collision to the detected vehicle in front of the ego-vehicle, and for lane departure warning. A similar application is Drivea [2], which provides obstacle collision warning, lane departure warning, and warning about speeding. A newer application, Movon FCW [3] is one of the few applications that can detect incoming cars, or cars that are not in full view, and can provide actual distance information.

In the research community, most of the recent obstacle detection efforts have been focused on sensors or sensory solutions that are able to deliver 3D data directly: radar, laser, or stereovision [4]. While some mobile devices are equipped with stereo cameras, which can be used for short distance accurate detection of obstacles [5], most devices are only capable of monocular vision.

Monocular obstacle detection faces multiple challenges, the most important being that there is no direct 3D information, and no direct, geometrical way of separating the obstacles from the road features, or the closer obstacles from the background. For the identification of the obstacle regions in the image, solutions include region segmentation [6,7], motion based analysis [8,9], or appearance analysis, either through heuristics such as symmetry [10,11], or through the use of machine learning [12,13]. For extracting the 3D position of the obstacle, monocular vision uses constraints imposed on the structure of the environment, such as the condition that the road is flat, which leads to the measurement of the points on the road by Inverse Perspective Mapping (IPM) [14,15].

The lack of 3D information considerably affects the robustness of an obstacle detection algorithm, and therefore most monocular applications make assumptions about the obstacles. Generic obstacle detection, independent of type or size, is much more difficult to achieve. While stereovision solutions, or other ranging solutions, can also employ assumptions about the obstacle’s nature, it also allows generic environment perception, such as in the form of dynamic occupancy grids [16], especially if the stereovision information is dense. The solution proposed in this paper comes to bring this power of generic environment perception to the monocular sensor as well, and it does so by using the dynamic particle-based occupancy grid as the common intermediate representation, a middle environment layer between the image data and the discrete obstacle layer. The use of a probabilistic, dynamic middle layer allows inference about the presence of obstacle parts in the scene, and about their motion, and also allows modeling the uncertainties of the sensing methodology.

In order to achieve a generic environment perception, a generic scene segmentation algorithm has to be employed. This paper presents such a solution, which is a refinement of a preliminary result of ours, based on IPM images, described in [17].

## 3. Solution Description

### 3.1. Overview

The process starts with the acquisition of an image from the mobile device’s camera. The image is then transformed into a bird’s eye view of the road by Inverse Perspective Mapping, using the camera calibration parameters. The IPM image is segmented along rays staring from the camera position, and a hypothetic obstacle map is generated. The obstacle map is used to update the particle based occupancy grid. In the update process, the vehicle motion information (speed and yaw rate) is obtained from the available sensors of the mobile device (GPS for speed, gyroscope or accelerometer for yaw rate). The occupancy grid attaches to each cell of the top view world an occupancy probability and a speed probability density.

**Figure 1:**
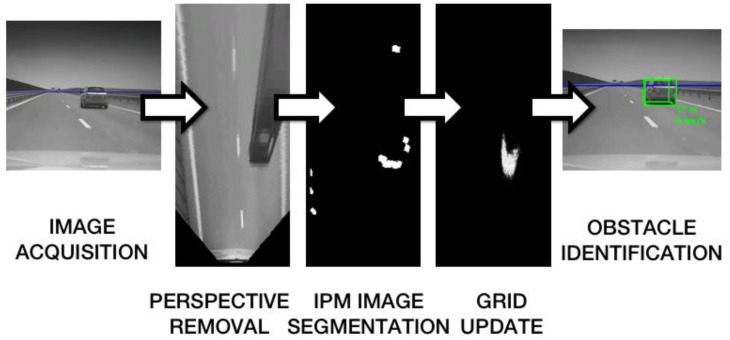
Overview of the obstacle detection process.

The estimated grid cells occupancy probabilities, and the speed vectors, are used in the process of obstacle extraction. The process is depicted in Figure 1.

### 3.2. Removing the Perspective Effect

**Figure 2:**
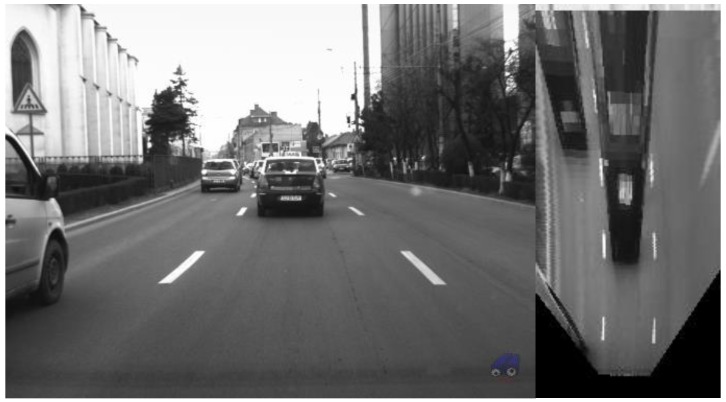
Removal of the perspective effect.

Assuming that the road is flat, the perspective image can be remapped in such a way that the features that belong to the road surface are displayed as they are seen from above and their lateral and longitudinal coordinates in the real world are proportional to the pixel coordinates (Figure 2).

In the IPM image, the obstacles are deformed, since they do not belong to the road surface and therefore their features do not obey the road geometry. For this reason, only the point of contact between the obstacle and the road is relevant for the next steps of the obstacle detection algorithm, as its position can be directly related to the position of the obstacle in the real world. The camera is assumed to be calibrated (the calibration methodology is described in Section 4), therefore the correspondence between any point in the 3D world and its corresponding projected pixel in the image plane is known. The 3D coordinate system is assumed to have the origin on the ground, below the camera, the x axis pointing to the driver’s right, the z axis indicating the direction of travel, and the y axis the height above the road (xOz) plane.

The removal of the perspective effect is performed using Algorithm 1:
**Algorithm 1:** IPM Transformation1 *Input:* Source image *I*2 *Output:* IPM image *I*_T_3 **For** each pixel of coordinates (*u*_T_, *v*_T_) of *I*_T_4 *x* = *k*
*u*_T_ + *x*_0_5 *z* = *j*
*v*_T_ + *z*_0_6 *y* = 07 (*u*, *v*) = Projection(*x*_W_, *y*_W_, *z*_W_)8 *I*_T_(*u*_T_, *v*_T_) = *I*(*u*, *v*)9 **End For**

The constants *k*, *j*, *x_0_* and *z_0_* are chosen in such a way that the most relevant portion of the road plane is displayed in the remapped image. The projection function uses the camera intrinsic and extrinsic parameters, which compose the projection matrix, for mapping a 3D point (expressed in the 3D world coordinates *x*, *y* and *z*), to the image plane.

The completely flat road assumption is restrictive, and the world will often not comply with it, due to the road geometry and, most often, to the balancing of the observer vehicle. This error is modeled in the grid measurement model used for grid state update.

### 3.3. Finding the Candidate Obstacle Regions

From the top view IPM image we have to identify the areas corresponding to the contact points between the obstacle and the road. Since we want our system to be as form-independent as possible, we will not use shape-based clues, such as symmetry.

**Figure 3:**
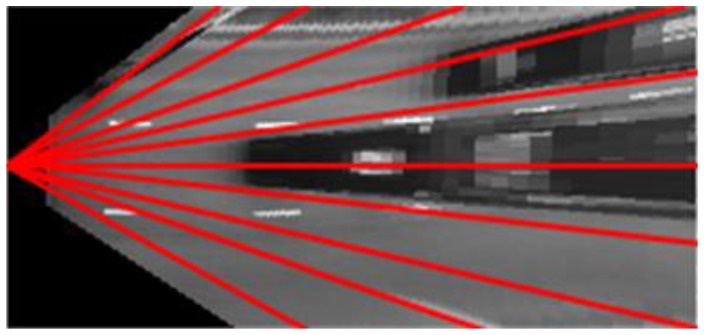
Scanning the Inverse Perspective Mapping (IPM) image with rays starting from the camera location.

In the IPM image, an upright obstacle has the tendency to expand radially from the camera. We use this property to scan the IPM image with rays for each angular degree of the field of view, with each ray converging on the camera position point (Figure 3).

For each scan ray, the angle and the minimum and maximum distance from the camera are known. If an obstacle is present on such a ray, its position along the ray must be found. As the obstacles do not comply with the flat road assumption of the IPM transformation, they will be deformed such that they will most likely stretch for the whole remainder of the ray. Thus, the only relevant information about the obstacle is its starting distance along the ray, the point where the obstacle touches the ground.

In order to find the obstacle distance along the scan ray, the grayscale values of the IPM image corresponding to the ray are recorded in a vector *g*_α_(*d*), *α* denoting the angle of the ray, and *d* being the distance from the camera. For each candidate obstacle distance *d*, the following values are computed:

(1) $$\mu_{\alpha ,P}(d)=\frac{1}{d-3-d\_\min_{\alpha }+1}\sum_{k=d\_\min_{\alpha }}^{d-3}g_{\alpha }(k)$$

(2) $$\mu_{\alpha ,D}(d)=\frac{1}{d\_\max_{\alpha }-d-3+1}\sum_{k=d+3}^{d\_\max_{\alpha }}g_{\alpha }(k)$$

(3) $$\mu_{\alpha ,M}(d)=\frac{1}{7}\sum_{k=d-3}^{d+3}g_{\alpha }(k)$$

These quantities are the mean intensity values for the proximal region ($\mu_{\alpha ,P}$), middle region ($\mu_{\alpha ,M}$) and distal region ($\mu_{\alpha ,D}$) of a ray of angle α with respect to the camera orientation, and the hypothetical obstacle distance *d*. Based on these mean values, we have to decide if a distance *d* may in fact be a distance to an obstacle touching the road. First, a binary function of *d* is designed, indicating whether the intensity profile of the ray of angle α supports the hypothesis of an obstacle being present at the distance *d*.

(4) $$\omega_{\alpha }(d)=\{\begin{array}{c} 1,\;if\;(\mu_{\alpha ,P}(d)-\mu_{\alpha ,M}(d)>\sigma )\;\mathrm{and}\;((\mu_{\alpha ,D}(d)-\mu_{\alpha ,M}(d)>\sigma )\mathrm{or}\;(\mu_{\alpha ,P}(d)-\mu_{\alpha ,D}(d)>\sigma ))\\ 0,\;otherwise \end{array}$$

In Equation (4), *σ* is the graylevel standard deviation of the IPM image, a measure of the image contrast. Intuitively, Equation (4) states that an obstacle point of contact with the road should be darker than the road leading to it, and also darker than the rest of the obstacle along the ray. The shadow area below a car passes this test. Darker areas that continue along the ray are also taken into consideration. Off course, this assumption is valid only during daytime, and will not be valid at night, so this solution is not aplicable at night.

If multiple candidate distances for a ray have $\omega_{\alpha }(d)$ > 0, the best candidate has to be selected. A continuous score function is defined:

(5) $$\varsigma_{\alpha }(d)={\left(\left|\mu_{\alpha ,P}(d)-\mu_{\alpha ,M}(d)\right|\cdot \left|\mu_{\alpha ,D}(d)-\mu_{\alpha ,M}(d)\right|\cdot \left|\mu_{\alpha ,P}(d)-\mu_{\alpha ,D}(d)\right|\right)}^{\frac{1}{3}}$$

**Figure 4:**
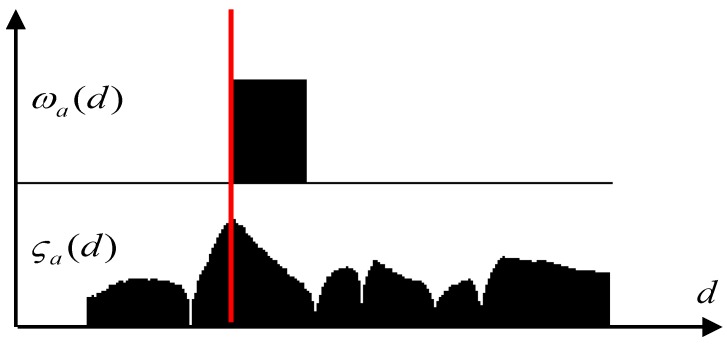
Finding the obstacle’s position along a ray.

Out of all the values *d* that have $\omega_{\alpha }(d)$ > 0, the one having a maximum $\varsigma_{\alpha }(d)$ is kept as the obstacle position. The process is depicted in Figure 4: on the first row the binary function $\omega_{\alpha }(d)$ is shown, and the second row depicts the continuous score function. The red line indicates the choice for the obstacle’s position.

**Figure 5:**
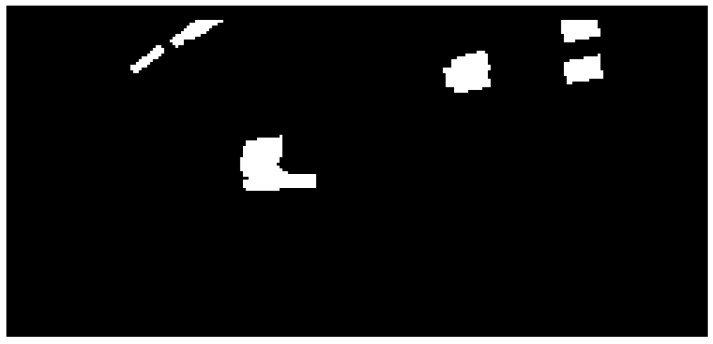
The measurement binary obstacle grid.

After the obstacles are located on each ray, a binary grid is constructed from these results (Figure 5).

### 3.4. The Particle-Based Occupancy Grid

The particle based occupancy grid is a freeform dynamic world representation, which tracks a bird’s eye grid view of the world, in terms of occupancy (presence of an obstacle) and speed vector probability density for each grid cell.

The dynamic occupancy grid depicts the obstacles as composed of particles having individual position and speed. Each discrete grid cell will thus contain a certain number of particles, each particle having its own speed vector. The occupancy probability of each grid cell is described by the number of particles in that cell (more particles, more likely the cell is occupied by an obstacle). The particles in this algorithm can be regarded in two ways: as hypotheses, such as in the case of particle filtering algorithms such as CONDENSATION [18], but also as actual building blocks of the environment, which can move from one cell to another in order to model the dynamic evolution of the environment (and, by doing this, achieve the prediction of the environment’s state).

The world is represented by a 2D grid, mapping the bird’s eye view 3D space into discrete 20 cm × 20 cm cells. The aim of the grid tracking algorithm is to estimate the occupancy probability of each grid cell, and the speed vector components on each axis (longitudinal and lateral).

Considering a coordinate system where the *z* axis points towards the direction of the ego-vehicle, and the *x* axis points to the right, the obstacles in the world model are represented by a set of particles:

(6) $$S=\{p_{i}|p_{i}=(c_{i},r_{i},vc_{i},vr_{i},a_{i}),i=1\ldots N_{S}\}$$

Each particle *i* has a position in the grid, described by the row *r_i_* (a discrete value of the distance in the 3D world *z*) and the column *c_i_* (discrete value of the lateral position *x*), and a speed, described by the speed components *vc_i_* and *vr_i_*. An additional parameter, *a_i_*, describes the age of the particle, since its creation. The purpose of this parameter is to facilitate the validation and the speed estimation process, as only particles that survive in the field for several frames are taken into consideration.

The total number of particles in the scene *N_S_* depends on the scene load (the number of obstacles in the field), and is not known a-priori, but updated each time a new obstacle cell is discovered by measurement. Having the population of particles in place, the occupancy probability of a cell *C* is estimated as the ratio between the number of particles whose position coincides with the position of the cell *C* and the total number of particles allowed for a single cell, *N_C_*.

(7) $$P_{O}(C)=\frac{\left|\{p_{i}\in S|r_{i}=r_{c},c_{i}=c_{c}\}\right|}{N_{C}}$$

The number of allowed particles per cell *N_C_* is a constant of the system. In setting its value, a trade-off between accuracy and time performance should be considered. A large number means that on a single cell multiple speed hypotheses can be maintained, and therefore the tracker can have a better speed estimation, and can handle fast moving objects better. However, the total number of particles in the scene will be directly proportional with *N_C_*, and therefore the speed of the algorithm will decrease. The current value for *N*_C_ is 50.

The speed of a grid cell can be estimated as the average speed of its associated particles, if we assume that only one obstacle is present in that cell.

(8) $$(vc_{C},vr_{C})=\frac{\sum_{p_{i}\in S,x_{i}=x_{c},z_{i}=z_{c}}\left(vc_{i},vr_{i}\right)}{\left|\{p_{i}\in S|r_{i}=r_{c},c_{i}=c_{c}\}\right|}$$

Thus, the population of particles is sufficiently representative for the probability density of occupancy and speed for the whole grid. Multiple speed hypotheses can be maintained simultaneously for a single cell, and the occupancy uncertainty is represented by the varying number of particles associated to the cell. Based on the world model, the environment tracking algorithm becomes the continuous creation, moving and destroying the particles, a process driven by the measurement information, which is the binary grid described in the previous section.

**Figure 6:**
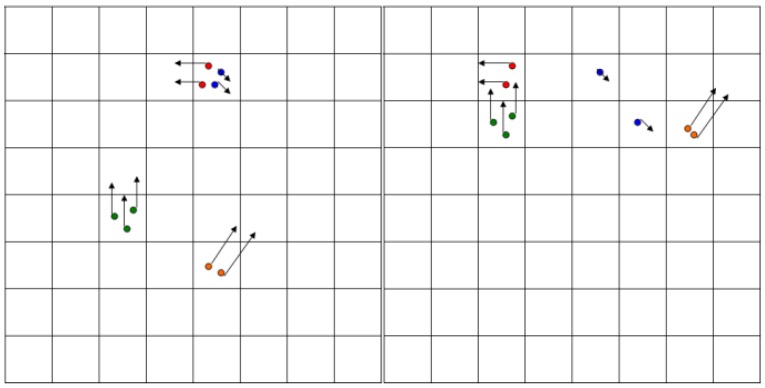
Migration of particles from one cell to another, as prediction is applied [16]. © 2011 IEEE. Reprinted, with permission, from IEEE Transactions on Intelligent Transportation Systems, Vol. 12, No. 4.

The first step of the algorithm is the *prediction*, which is applied to each particle in the set. The positions of the particles are altered (see Figure 6) according to their speed (or we can say that the particles move according to their own speed vector), and to the motion parameters of the ego vehicle (speed and yaw rate), read from the mobile device’s sensors, GPS and accelerometer/gyroscope. In addition, a random amount is added to the position and speed of each particle, for the effect of stochastic diffusion, modelling the uncertainty of the motion model with respect to the real-world movement of the obstacles. The second step is the *processing of measurement* information. This step is based on the raw occupancy cells provided by processing the IPM image, and provides the measurement model for each cell.

**Figure 7:**
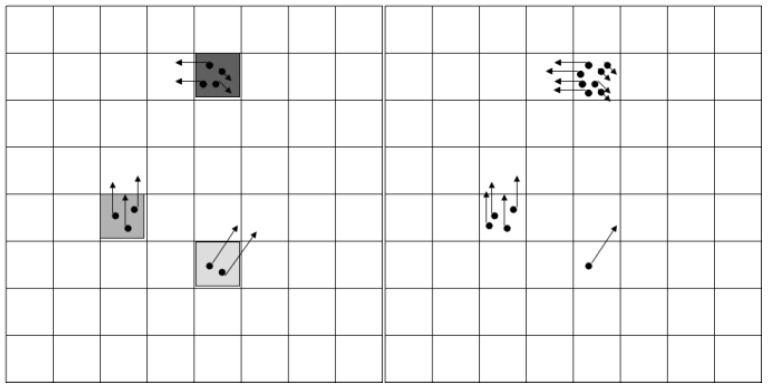
Weighting and resampling: The weight of the occupied hypothesis is encoded in the darkness of the cell of the left grid. In the right grid, the effect of resampling is shown, as particles are multiplied or deleted [16]. © 2011 IEEE. Reprinted, with permission, from IEEE Transactions on Intelligent Transportation Systems, Vol. 12, No. 4.

The measurement model information is used to *weight* the particles, and *resample* them in the same step (see Figure 7). By weighting and resampling, the particles in a cell can be multiplied or reduced. The final step is to estimate the occupancy and speeds for each cell. More details about the grid tracking process are provided in our previously published work [16].

### 3.5. Updating the Dynamic Occupancy Grid from the IPM-Based Measurement

The update process uses the measured binary grid, and the uncertainty associated to the creation of this grid, in the process of adjusting the particle population. In our previously published work, the binary grid was created from a digital map generated by processing dense stereovision data. The position uncertainty of an obstacle cell is derived from the uncertainty of the stereovision process. The standard deviation of a distance *z* estimated by a stereovision system of baseline *b* and focal length *f*, having a matching uncertainty of $\sigma_{d}$ pixels, is:

(9) $$\sigma_{z}=\frac{z^{2}\sigma_{d}}{bf}+\sigma_{z0}.$$

The standard deviation of the other grid coordinate, the lateral position *x*, is:

(10) $$\sigma_{x}=\frac{x\sigma_{z}}{z}+\sigma_{x0}.$$

The lateral position uncertainty depends on the distance uncertainty, which is the most important uncertainty of the measurement process. If we want to adapt the occupancy grid tracking mechanism to use binary measurement grids generated by processing IPM images, the computation of distance uncertainty has to be adapted to the IPM method of distance computation.

**Figure 8:**
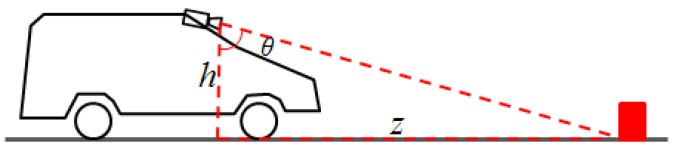
The principle of IPM distance measurement.

The process of estimating the distance of an obstacle from the camera when using IPM images is described in Figure 8. The height of the camera is known, along with its orientation. A pixel in the image corresponds to a line from the camera, which makes with the vertical the angle θ. This angle depends on several factors: the angle between the camera’s optical axis and the vertical, the position of the pixel in the image plane, the focal length of the camera, and the position of the principal point. We are not concerned with these details. For all practical purposes, we can say that we know θ, and thus, from the red triangle described in Figure 8, we can compute *z*.

The IPM image is not constructed by using angles and heights, but by using the projection matrix, as described in Section 3.2. However, the simplified model is useful in understanding the mechanism of uncertainty. As the height of the camera above the ground can be considered, to a reasonable degree, to be fixed, the uncertainty affects mostly the angle θ. This uncertainty comes from the pitching of the observing vehicle, as the car encounters uneven surfaces, accelerates or brakes. Small and temporary deviations of the real road surface from the flat road assumptions can also be modeled as errors in the θ angle, while long term, significant deviations cannot be modeled and will lead, eventually, to wrong estimations. An experimentally tuned value of σ_θ_ = 0.25° was chosen to account for these situations.

The relation between *z* and θ is:

(11) z=htanθ.

The relation between the uncertainty of *z* and the uncertainty of θ is:

(12) $$\sigma_{z}=\frac{dz}{d\theta }\sigma_{\theta }.$$

This can be written as:

(13) $$\sigma_{z}=h(1+\tan^{2}\theta )\sigma_{\theta }.$$

Using Equation (11), we can replace the tangent, and obtain the final equation for the uncertainty of *z*:

(14) $$\sigma_{z}=h\left(1+\frac{z^{2}}{h^{2}}\right)\sigma_{\theta }+\sigma_{z0}.$$

The quantity $\sigma_{z0}$ is a constant covering other sources of uncertainty that cannot be modeled analytically.

For each cell in the grid, the difference *d*_z_ in grid row coordinates and the difference *d*_x_ in grid column coordinates is computed between the position of the grid cell and the position of the nearest obstacle cell in the binary obstacle map segmented from the IPM image. Using these distances and the uncertainty values computed from Equations (14) and (10), we can compute the measurement probability density under the assumption of the cell being occupied, by employing the bivariate Gaussian function:

(15) $$p_{occupied}=\frac{1}{2\pi \sigma_{z}\sigma_{x}}e^{-\frac{1}{2}\left({\left(\frac{d_{z}}{\sigma_{z}}\right)}^{2}+{\left(\frac{d_{x}}{\sigma_{x}}\right)}^{2}\right)}$$

Based on the measurement probability value, the predicted particles in a cell may be multiplied or decimated.

The particle creation, movement, multiplication and destruction mechanisms of the dynamic occupancy grid [16] can now be used with the IPM binary obstacle grid as measurement data.

### 3.6. Detection of Individual Obstacles

The dynamic grid’s cells that hold a significant number of particles are considered occupied, and therefore parts of obstacles. A grouping algorithm, which takes into account the proximity of occupied cells, but also the agreement of their speed vectors, extracts connected regions from the grid, regions that describe individual objects. An oriented rectangle shape is matched to these connected regions, and the 3D object is extracted as a cuboid (obviously, the height of the object above the road cannot be computed, and a fixed value is set).

Due to the nature of the occupancy grid, its cells will have a speed vector, computed as the resultant of the individual speed vectors of the particles. After the grid cells are grouped into individual obstacles, a resultant speed vector for the cell group is computed, and this vector is assigned to the detected object.

**Figure 9:**
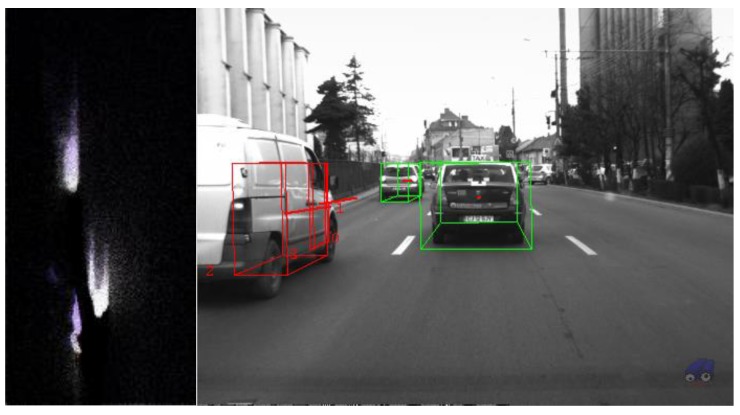
Obstacle detection results: (**Left**) the dynamic occupancy grid; and (**Right**) the extracted cuboids.

The resulted objects have length, width, orientation and speed, and can be therefore classified into static and dynamic. The process is seen in Figure 9, where the stationary vehicles are shown in green, and the moving vehicle is shown in red.

## 4. Implementation for Android Mobile Devices

### 4.1. Software Architecture of the Solution

**Figure 10:**
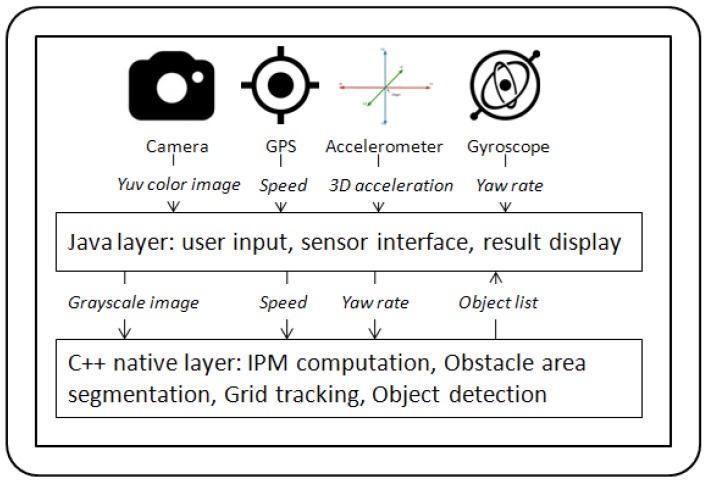
Architecture of the solution.

The solution was implemented as an Android application, using Java for the user interface, and native C++ for the algorithm processing workload (Figure 10).

The calibration process is simplified by the automatic retrieval of the camera’s horizontal and vertical field of view angles using the Android API. The fixed image resolution of 640 × 480 pixels, combined with the known field of view, give us the focal distance in pixels. The position of the principal point is assumed to be in the center of the image. The user is asked to tune the pitch angle and the camera height, while the IPM image is shown with a distance grid superimposed, so that the user can adjust these parameters until top view scene is in accordance to the grid.

The grid tracking algorithm requires the motion parameters of the host vehicle, the speed and the yaw rate. The speed is provided by the mobile device’s GPS receiver. For the yaw rate, the on-board gyroscope is used when available; otherwise, the lateral acceleration combined with the speed is used to infer the yaw rate.

### 4.2. Calibration

#### 4.2.1. Calibration of the Intrinsic Camera Parameters

The intrinsic parameters of the camera are the focal length (usually expressed in pixels), the position of the principal point (the intersection between the optical axis and the image plane), and the distortion coefficients. All these parameters can be computed using a calibration toolbox [19]. However, using a calibration toolbox requires a certain expertise, a significant amount of effort from the part of the user, and makes the system hard to set up and use.

An alternative is to make several simplifying assumptions, and use the information provided by the Android API. We can assume that the principal point is in the center of the image (modern camera technology ensures that the principal point is close to the image center, and a precise estimation of this point is not required for monocular applications), and to ignore the distortion coefficients of the lens. The only intrinsic parameter that still needs to be calibrated is the focal distance.

Using the available Android API, developers can access the vertical and the horizontal view angles, by calling the *getVerticalViewAngle*() or the *getHorizontalViewAngle*() methods of the Camera. Parameters object. Denoting the horizontal view angle by α, and the image width in pixels by *w*, the focal distance in pixels *f* can be computed as:

(16) $$f=\frac{w}{2\tan(\alpha /2)}$$

The same equation can be applied for the vertical field of view, and the image height *h*, and the resulted focal length should be the same, if the camera pixel is square. If the camera supports variable zoom, the focal length can be adjusted to take into account the zoom level. Using Equation (16), the intrinsic parameters of the camera can be computed automatically when the system is started.

The intrinsic parameters matrix **A** is thus expressed by Equation (17), which assumes that the principal point of the camera model is in the image center:

(17) $$A=\left(\begin{array}{ccc} f & 0 & w/2\\ 0 & f & h/2\\ 0 & 0 & 1 \end{array}\right)$$

In order to test the automatic approach for focal distance calibration, we have performed classic calibration, using the Open CV calibration toolbox [19], for several mobile devices. The results are shown in Table 1.

While not perfectly identical, the results are in good agreement, with less than 4% percent error. These values can be used in a monocular vision application.

#### 4.2.2. Calibration of the Extrinsic Camera Parameters

**Figure 11:**
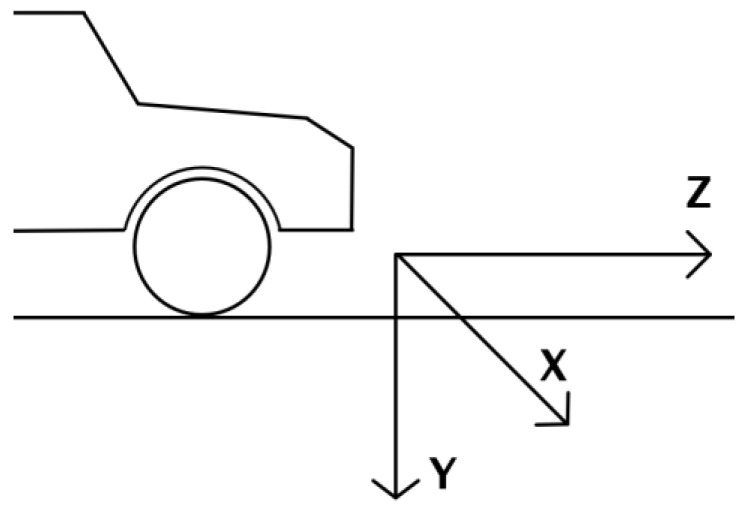
The coordinate system of the vehicle.

The extrinsic parameters relate the 3D coordinate system of the camera to the 3D coordinate system of the vehicle. The detection is performed in the coordinate system of the host vehicle, which is has the origin on the longitudinal symmetry plane of the car, whose intersection with the road plane is the distance axis, *Z*. The lateral coordinate, *X*, points to the right of the direction of travel, and the *Y* coordinate points towards the road. The position of the origin coincides with the closest point of the road in front of the vehicle, as seen in Figure 11.

The extrinsic parameters are represented by the rotation matrix **R** and the translation vector **T**. A full calibration of these parameters can be performed using a calibration toolbox, and a reference scene with objects at known positions in the car coordinate system. Unfortunately, this means again that the system cannot be set up easily. Instead, we have made several simplifying assumptions, and designed a simple interface for allowing the user to easily tune a simplified extrinsic parameter set.

The user has to set up the mobile device in the vehicle, aligning the camera with the longitudinal axis of the car as best as possible. This alignment is not critical, and can be easily achieved by the user. The calibration view of the application allows the user to input the height of the camera above the ground (*H*), and the distance from the camera to the front end of the car (*L*). These parameters can be easily measured. A very critical parameter is the pitch angle *θ*, which must be also input by the user.

**Figure 12:**
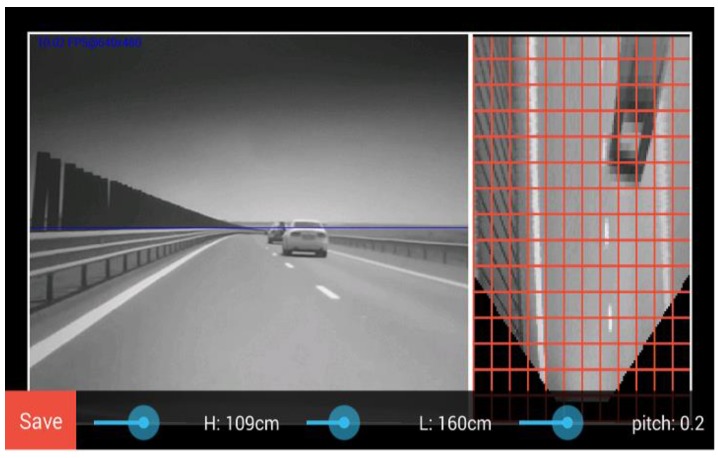
The calibration user interface.

To assist the user, the system performs live Inverse Perspective Mapping on the capture images, in real time. A grid is superimposed on the IPM result, and the horizon line, resulted from the pitch angle, is projected on the perspective image, as seen in Figure 12. This way, the user can adjust the parameters until the horizon line matches the true horizon of the observed scene, and if the observed scene has objects at known distances, these distances can be compared with the metric grid during parameter tuning. If road markings are visible, a good indication for the correctness of the pitch angle is that they must appear parallel in the IPM image.

The parameters *H*, *L* and *θ* input by the user are used to generate the translation vector and the rotation matrix:

(18) $$T={\left(\begin{array}{ccc} 0 & H & L \end{array}\right)}^{T}$$

(19) $$R=\left(\begin{array}{ccc} 1 & 0 & 0\\ 0 & \cos\theta & -\sin\theta \\ 0 & \sin\theta & \cos\theta \end{array}\right)$$

Now the intrinsic and the extrinsic parameters can be used to build the projection matrix, which will relate any 3D point in the car coordinate system to a point in the image. This matrix is used for computing the Inverse Perspective Transform image.

(20) P=A[R|T]

The user calibration user interface can be extended easily to include sliders for the other two rotation angles, yaw and roll. Each of these angles will create a rotation matrix, which can be multiplied to the rotation matrix of the pitch, to form the total rotation. However, the user can position the mobile device to a reasonably accurate heading (yaw), and level it to a reasonable roll angle. Errors of the order of magnitude of degrees (±5°) in these angles will have a certain effect on the final results, causing the obstacles to become slightly misaligned, or having their lateral position slightly off, but errors of much less magnitude in the pitch angle will have significant effects in distance estimation. For this reason, and for trying to keep the user interface as simple as possible, the compromise of having the user tune only the pitch angle was made.

Now the intrinsic and the extrinsic parameters can be used to build the projection matrix, which will relate any 3D point in the car coordinate system to a point in the image. This matrix is used for computing the Inverse Perspective Transform image.

## 5. Experimental Setup and Evaluation

**Figure 13:**
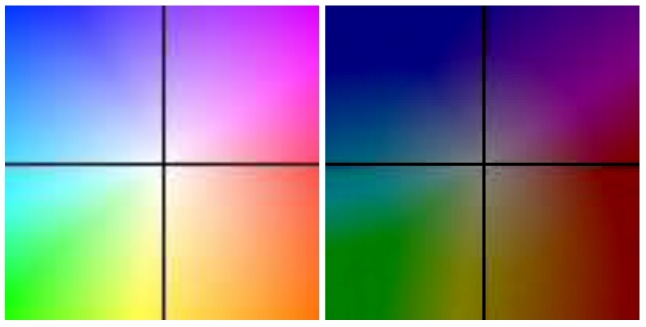
Color coding for occupancy grid speed vectors (full and half occupancy). The hue represents the direction (i.e., red means the obstacle cell is moving to the right), the saturation represents the speed magnitude, and the intensity represents the occupancy probability of the grid [16]. © 2011 IEEE. Reprinted, with permission, from IEEE Transactions on Intelligent Transportation Systems, Vol. 12, No. 4.

**Figure 14:**
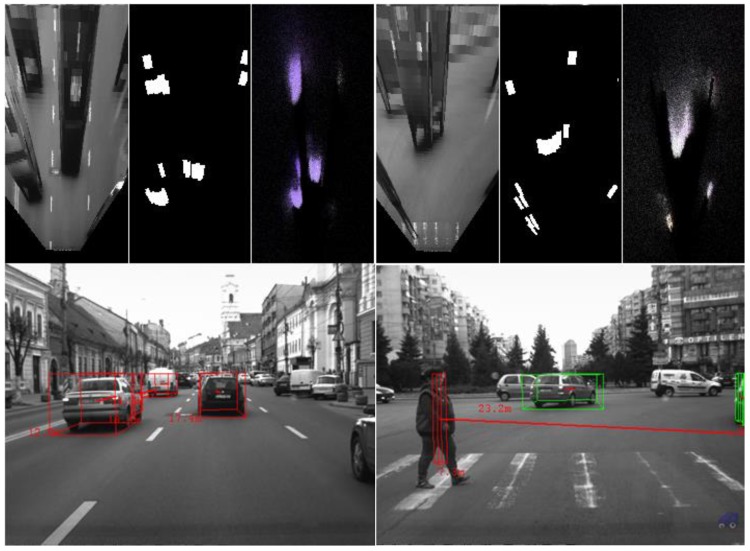
(**Left**) Three dynamic objects, going in the same direction as the observing vehicle; and (**Right**) pedestrian on a worn out crossing. In the distance, a vehicle is detected as stationary, as it waits to enter the roundabout. The IPM image, the segmentation result and the occupancy grid estimation are shown on top of the perspective image.

**Figure 15:**
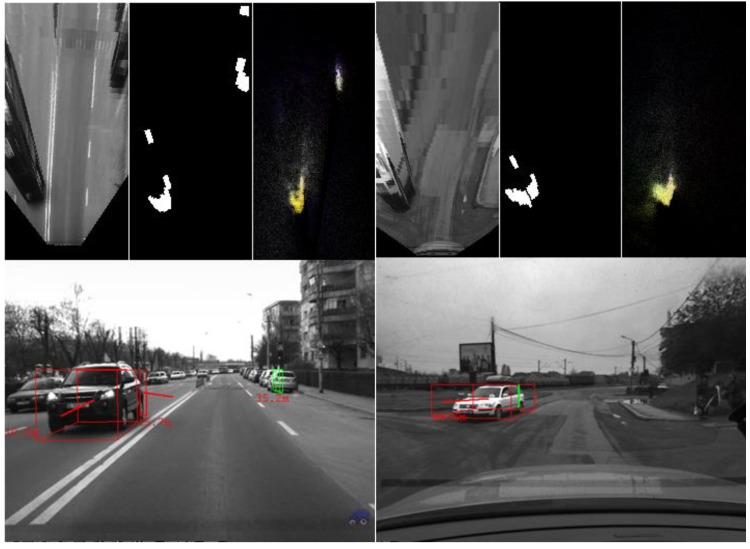
(**Left**) Detected incoming vehicle, at a relative speed of around 100 km/h; and (**Right**) diagonally crossing object, on patched road. The uneven road surface does not impede detection.

**Figure 16:**
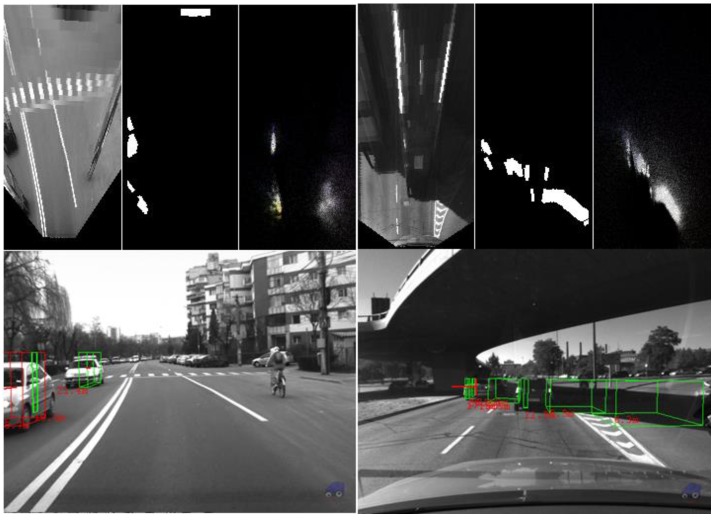
Failure cases: (**Left**) The bicyclist on the right side lane is not detected, due to its low contrast between the wheels and the road; and (**Right**) false positives due to strong shadows in very bright light.

The algorithm was tested on recorded sequences from mostly urban scenarios, under a various range of lighting conditions. Obstacles from a wide range of shapes, speeds and orientations have been successfully detected, and their static and dynamic properties successfully measured. The IPM image, the binary obstacle grid, the estimated occupancy grid with color coding of speed and orientation for each cell (see Figure 13), and the projected cuboids on the original perspective image are shown for several real traffic scenarios, in Figure 14, Figure 15 and Figure 16.

In Figure 14 and Figure 15, successful situations are shown in a variety of challenging scenarios, and two clear failure cases are shown in Figure 16. The scenarios are explained in the captions of the figures. The detected moving objects are displayed with the color red, and the stationary objects with green.

The qualitative analysis shows that most of the obstacles are correctly detected, but there are still problems with objects of low contrast (especially at the point of contact with the road, which is the point that matters for IPM), and with strong shadows in bright daylight. A video showing results for a 11 min drive sequence (recorded and processed offline) can be seen at http://vimeo.com/107588262. Obstacles on the road are detected at various distances, up to 40 m. Each obstacle is bounded by a cuboid and the distance from the ego-vehicle to the obstacles is written below the cuboid. Stationary obstacles are shown as green cuboids, while moving obstacles are shown as red cuboids. On the top left of the frame, the occupancy grid is displayed, using the color coding for speed and orientation of each cell, as described by Figure 13.

The system was tested on multiple mobile devices: the Samsung Galaxy S5 phone, and the LG GPad 8.3 and the Samsung Galaxy Tab Pro tablets. Tests were performed in city traffic with the devices plugged in, allowing them to run at full processing power. The usage of vocal assistance and feedback will reduce the necessity of the device screen to be on. Depending on the complexity of the scene, the system’s processing time ranged from 50 to 80 ms. The resulted frame rate ranged between 9 and 15 fps, as the whole frame cycle includes the acquisition process and the result display. A frame rate comparison, using the same recorded video, is presented in Table 2.

In order to assess the battery consumption of the system, we have conducted power tests with the devices running on battery power, during 20 min runs, and the overall battery drain was measured. The results are presented in Table 3. Battery consumption is also influenced by the fact that currently the output of the system is visual, requiring the device screen to be on all the time. However, the real use of such a driving assistance system will be either as an audio warning system, or as a data source for other devices (such as the car’s computer itself), connected into an Internet of Things configuration, and the need for the screen to be on all the time will disappear.

**Figure 17:**
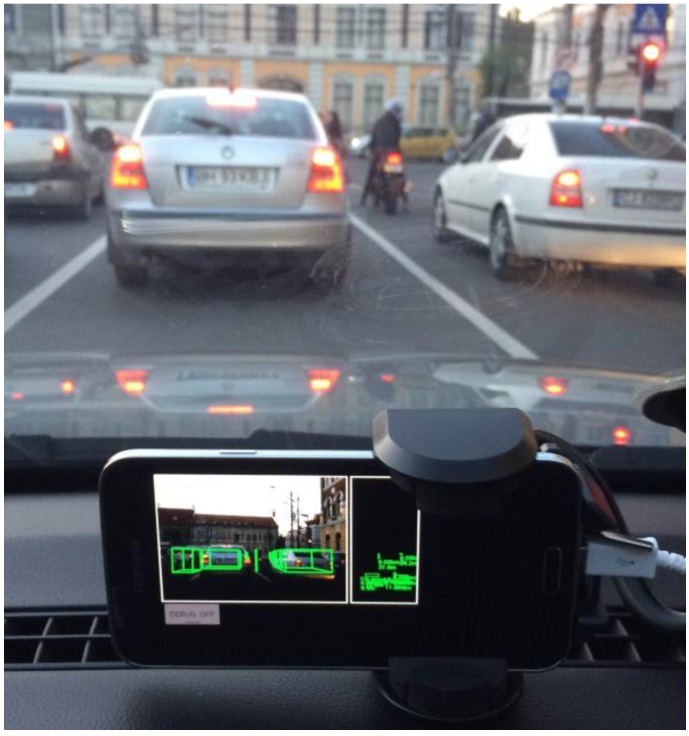
Obstacle detection on the Samsung Galaxy S5 smartphone: Stop and go traffic.

**Figure 18:**
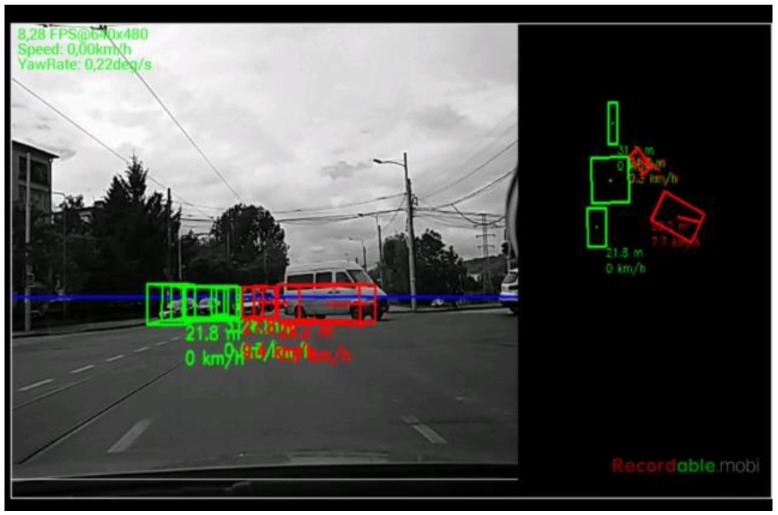
Obstacle detection on the Samsung Galaxy Tab Pro, waiting to cross an intersection: (**Left**) perspective projection of the detection results; and (**Right**) top view of the detected obstacles.

**Figure 19:**
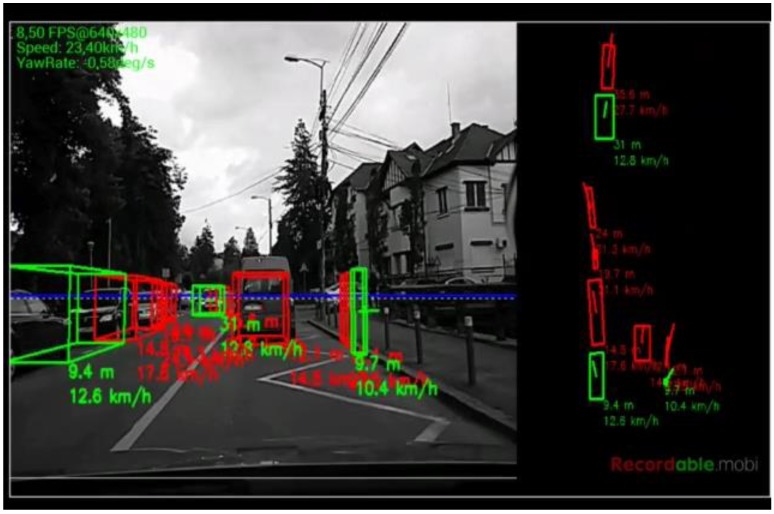
Detection of moving and parked obstacles. The speed vector of the parked vehicles is not correctly estimated as their limits in the direction of our motion are not clear.

Figure 17, Figure 18 and Figure 19 show the application running on mobile devices, in urban traffic.

**Figure 20:**
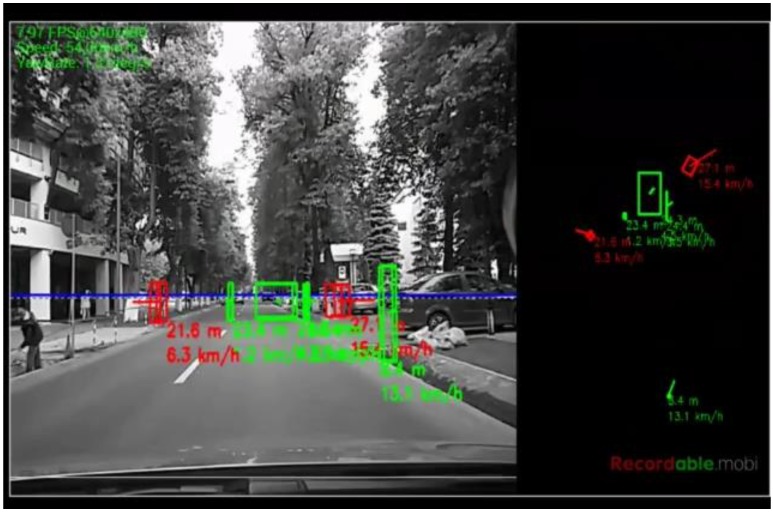
False positives due to shadows on the road.

Due to the monocular, grayscale nature of the information used for detection, the results are sometimes affected by false positives, due to shadows on the road (Figure 20), or by false negatives when the contrast between the road and the obstacle is not high enough.

In order to assess the accuracy of the obstacle measurement process, the obstacle range obtained from the proposed solution is compared with the range provided by a stereovision-based obstacle tracking system. The monocular solution was tested on the left frame of the stereo pair, and the stereo system used both images. The stereo images were acquired with a 22 cm baseline stereo rig, using two 2/3” CCD cameras (8.3 μm × 8.3 μm pixel size) equipped with 8 mm lenses, the images being down sampled to a resolution of 512 × 383 pixels. This stereovision system is a custom rig, built for accuracy, not a stereo system of a mobile device, which have a too small baseline to be used for measuring distances in the ranges required for the driving environment.

An obstacle in front of the observing vehicle was tracked form approximately 800 frames, or about 80 s. During this time, the target vehicle increased and decreased its distance from the observer multiple times, and the observing vehicle was following. The target vehicle was not knowledgeably involved in the experiment, and its actions were independent. The track was lost when the target vehicle passed beyond 35 m, which seems to be the detection limit of our system.

**Figure 21:**
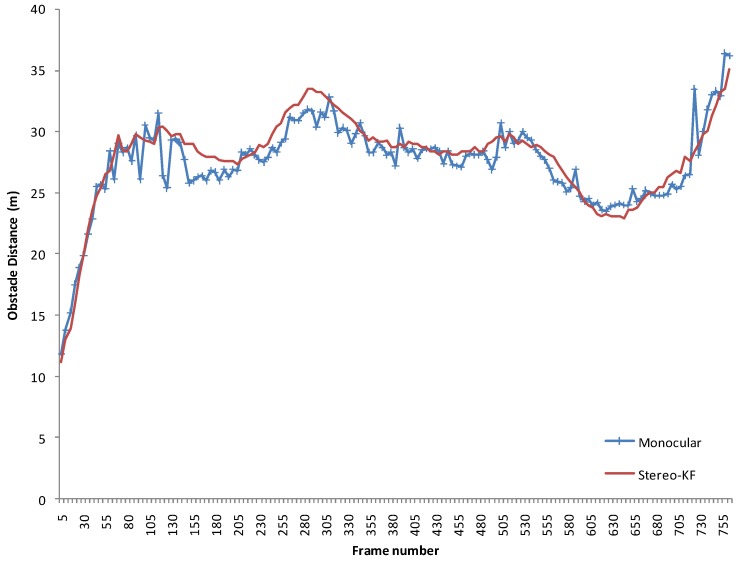
Distance estimation comparison between the proposed monocular solution and a stereovision-based solution.

The range estimation results are shown in Figure 21. While noisier, the estimations of the monocular system follow closely the stereovision results. For the whole sequence, the Root Mean Square Error was 1.33 m, and the Mean Average Error 1.02 m.

In order to estimate the detection rate, we have used the sequence presented in the video available at the link http://vimeo.com/107588262, for which we have also the right image of the stereo pair, and therefore a comparison between the monocular and the stereovision method can be performed. A manual identification of the objects in the distance range of 0 to 40 m, and spanning a lateral distance of three lanes (the current lane and two side lanes), was performed on 40 frames of the sequence, equally spaced in time at approximately 15 s, covering a driving time of 11 min. The obstacles were heterogeneous, including cars, pedestrians, and scenery. The detection results are presented in Table 4. As expected, the monocular solution shows a poorer detection rate performance compared with stereovision, but this rate was computed taking into account all types of obstacles, including scenery such as poles, which have a low contrast with respect to the road surface, and also the obstacles which are only partially visible in the image, such as overtaking/overtaken cars, which are easily detected by stereovision, but cannot be reliably detected by the monocular solution. If the comparison were limited to only cars and pedestrians clearly visible in front of the vehicle (at risk of being hit), the detection rates would be much more similar.

The distance measuring capabilities of the monocular application was also tested against ground truth data acquired with laser measuring equipment. In the absence of a laserscanner, distances to stationary targets were measured using a Leica Disto D5 laser ranging tool, having a measurement of error of ±1 mm, and an effective range of 0.05 m to 200 m. The mobile application was run on a Samsung Galaxy S6 mobile phone. A comparison between the distance reported by the monocular mobile application for the detected objects, and the ground truth from laser measurements is provided in Table 5. The relative error of distance measurement is around 10%, which is expected from a monocular vision system.

## 6. Conclusions and Future Work

This paper describes a mobile device based, monocular computer vision solution for detecting the position, size and speed of the obstacles in traffic. The detection method uses a simple and fast segmentation technique, followed by free-form tracking of the obstacle areas using the particle based occupancy grid. The tracked grid cells are grouped into discrete obstacles. As the grouped cells already have a speed vector, the resulted obstacles will have speed vector as well, with magnitude (absolute speed) and orientation. While most existing solutions are able to detect clearly visible vehicles, especially those that are followed by the host vehicle, the proposed system makes little assumptions about the appearance of the obstacles.

The system was tested in real, heavy urban traffic, and shows promising results, turning a mobile device into a driving assistance sensory system.

There is significant room for improvement. Due to monocular vision limitations, the false positives or the false negatives are always a threat, especially for a generic obstacle detection system. The reduction of the error rate can be achieved by using color data (as they are already provided by the device’s camera) or optical flow. The integration of a lane tracker algorithm to the system is in progress, and the estimated pitch angle of the lane can reduce the distance measurement errors caused by forward-backward balancing.

In order to transform the method into a useful system for driving assistance, the detection results have to be communicated non-disruptively. A basic audio warning signal can be issued when collision is likely, but a more detailed description of the obstacles, including all their parameters, can be transmitted using Bluetooth or WiFi, to be used by other systems which may take active steps to prevent accidents, log data for traffic analysis or real time mapping, or monitor the driver’s behavior.

## Figures and Tables

**Table 1:** Focal distance calibration comparison.

	Samsung Galaxy S5	Sony Xperia Z1	Motorola Moto G	HTC One Mini 2
*f* computed using Android API	697	484	598	530
*f* computed using Calibration Toolbox	686	502	617	520

**Table 2:** Frame rate comparison using offline processing of a recorded sequence.

	Samsung Galaxy S5	Sony Xperia Z1	Motorola Moto G	HTC One Mini 2
*Average frame rate*	17.6	11.4	8.7	14.5

**Table 3:** Battery drain on a 20 min test run.

	Samsung Galaxy S5	Samsung Galaxy S6	Motorola Moto G	HTC One M8
*Battery usage (% of total)*	13%	11%	13%	12%

**Table 4:** Detection rate comparison between the monocular approach and stereovision.

	Ground Truth	Stereovision: True Positives	Monocular: True Positives	Stereovision: False Positives	Monocular: False Positives
*Number of obstacles*	162	157	110	6	3
*Detection rates*	-	96.7%	67.9%	3.7%	1.8%

**Table 5:** Distance measurement accuracy—comparison with laser data.

Ground Truth (m)	Monocular Estimated Distance (m)	Absolute Error (m)	Relative Error (%)
6.29	6.7	0.41	6.52%
11.87	11.6	0.27	2.27%
13.95	14.0	0.05	0.36%
14.08	15.7	1.62	11.51%
14.32	16.1	1.78	12.43%
17.32	18.1	0.78	4.5%
18.89	17.3	1.59	8.42%
22.81	21.7	1.11	4.87%
31.32	33.9	2.58	8.24%
31.34	34.7	3.36	10.72%

## References

[1] iOnRoad iOnRoad Augmented Driving Pro. http://www.ionroad.com/.

[2] Drivea Drivea—Driving Assistant App. http://www.appszoom.com/android_applications/transportation/drivea-driving-assistant-app_bdwmk.html.

[3] Movon Corporation Movon FCW. https://play.google.com/store/apps/details?id=com.movon.fcw.

[4] Sivaraman S., Trivedi M.M. (2013). Looking at Vehicles on the Road: A Survey of Vision-Based Vehicle Detection, Tracking, and Behavior Analysis. IEEE Trans. Intell. Transp. Syst..

[5] Petrovai A., Costea A., Oniga F., Nedevschi S. Obstacle detection using stereovision for Android-based mobile devices. Proceedings of the 2014 IEEE 10th International Conference on Intelligent Computer Communication and Processing.

[6] Haltakov V., Belzner H., Ilic S. Scene Understanding from a Moving Camera for Object Detection and Free Space Estimation. Proceedings of the 2012 IEEE Intelligent Vehicles Symposium.

[7] Wybo S., Tsishkou D., Vestri C., Abad F., Bougnoux S., Bendahan R. Monocular vision obstacles detection for autonomous navigation. Proceedings of the IEEE/RSJ International Conference on Intelligent Robots and Systems (IROS).

[8] Reisman P., Mano O., Avidan S., Shashua A. Crowd detection in video sequences. Proceedings of the 2004 IEEE Intelligent Vehicles Symposium.

[9] Zhang X., Jiang P., Wang F. Overtaking Vehicle Detection Using A Spatio-temporal CRF. Proceedings of the 2014 IEEE Intelligent Vehicles Symposium.

[10] Graefe V., Efenberg W. A Novel Approach for the Detection of Vehicles on Freeways by Real-Time Vision. Proceedings of the 1996 IEEE Intelligent Vehicles Symposium.

[11] Lim K.H., Ang L.M., Seng K.P., Chin S.W. Lane-Vehicle Detection and Tracking. Proceedings of the 2009 International Multi Conference of Engineers and Computer Scientists (IMECS).

[12] Sivaraman S., Trivedi M.M. (2010). A General Active-Learning Framework for On-Road Vehicle Recognition and Tracking. IEEE Trans. Intell. Transp. Syst..

[13] Arrospide J., Salgado L., Marinas J. HOG-like Gradient-based Descriptor for Visual Vehicle Detection. Proceedings of the 2012 IEEE Intelligent Vehicles Symposium.

[14] Bertozzi M., Broggi A. (1998). GOLD: A Parallel Real-Time Stereo Vision System for Generic Obstacle and Lane Detection. IEEE Trans. Image Process..

[15] Tuohy S., O’Cualain D., Jones E., Glavin M. Distance Determination for an Automobile Environment Using Inverse Perspective Mapping in OpenCV. Proceedings of the 2010 IET Signal and Systems Conference (ISSC).

[16] Danescu R., Oniga F., Nedevschi S. (2011). Modeling and Tracking the Driving Environment with a Particle-Based Occupancy Grid. IEEE Trans. Intell. Transp. Syst..

[17] Itu R., Danescu R. An Efficient Obstacle Awareness Application for Android Mobile Devices. Proceedings of the IEEE 10th International Conference on Intelligent Computer Communication and Processing (ICCP).

[18] Isard M., Blake A. (1998). CONDENSATION—Conditional density propagation for visual tracking. Int. J. Comput. Vis..

[19] OpenCV Camera Calibration with OpenCV. http://docs.opencv.org/doc/tutorials/calib3d/camera_calibration/camera_calibration.html.

